# Bluetongue disease in small ruminants in south western Ethiopia: cross-sectional sero-epidemiological study

**DOI:** 10.1186/s13104-018-3222-z

**Published:** 2018-02-08

**Authors:** Temesgen Abera, Molalegne Bitew, Debebe Gebre, Yosef Mamo, Yosef Deneke, Sukdeb Nandi

**Affiliations:** 1 Jimma University, College of Agriculture and Veterinary Medicine, Jimma, Ethiopia; 2 Ethiopian Biotechnology Institute, P.O. Box: 32853, Addis Ababa, Ethiopia; 3 Virus Laboratory, Center for Animal Disease Research and Diagnosis (CADRD), Indian Veterinary Research Institute (IVRI), Izatnagar, Uttar Pradesh, 243 122 India

**Keywords:** Bluetongue, c-ELISA, Risk factors, South West Ethiopia, Seroprevalence, Small ruminants

## Abstract

**Objective:**

The status of bluetongue disease, vectors for transmission of the disease and the serotypes involved are not clearly known in Ethiopia. This sero-epidemiological study was conducted to determine the seroprevalence and associated risk factors of bluetongue in small ruminants of South Western Ethiopia.

**Result:**

422 serum samples were screened for the presence of bluetongue virus (BTV) specific antibodies using competitive enzyme-linked immunosorbent assay (c-ELISA) and 30.6% (129/422) (confidence interval CI 26.2–35%) of the sheep and goat serum samples were found positive. Multivariate analysis of several risk factors like age, sex, altitude, body condition and species of animals were studied and it was observed that species of animals, age and altitude had significant influence (P < 0.05) on seropositivity to BTV. Goats showed more seropositivity to bluetongue as compared to sheep [AOR = 2.4, 95% CI (1.5–3.9), P = 0.001], adult animals were more seropositive [AOR = 3.1, 95% CI (1.9–5.1), P = 0.001] than other age groups and animals at the lowland [AOR = 3.1, 95% CI (1.5–6.4), P = 0.002] showed more seropositivity to bluetongue than midland and high land. Sex and body condition of the animals had no statistically significant (P > 0.05) effect on seropositivity to bluetongue.

## Introduction

Bluetongue (BT) is an infectious and non-contagious arthropod borne viral disease of domestic and wild ruminants [1]. Bluetongue virus is a member of the genus *Orbivirus* in the family *Reoviridae.* Its genome consists of ten double-stranded (ds) RNA segments coding for seven structural proteins (VP1–VP7) and four non-structural proteins (NS1–NS3 or NS3A, and NS4). At present 27 serotypes have been reported throughout the world [2, 3].

Bluetongue is endemic primarily in the tropical and subtropical regions between the latitudes of 40^o^N and 35^o^S where vectors (*Culicoides* spp.) are present. In Africa, Bluetongue has been known in South Africa for over a hundred years [4] and to date 21 of the 27 known BTV serotypes have been isolated from sheep [5]. The major vector in Africa is considered to be *C. imicola.* The incursion of BTV serotype 2 into North Africa (Algeria, Libya, Morocco and Tunisia) has been confirmed in 2004 [6]. However, in Eastern Africa the information about the prevalence of bluetongue is still unclear. Toye et al. [7] in western Kenya reported the highest seroprevalence (94.2%) of bluetongue in cattle and detected nine different serotypes (BTV 1, 3, 7, 12,15,16, 19, 22 and 24) by real time PCR.

Ethiopia is endowed with huge livestock population including 53.99 million cattle, 25.5 million sheep and 24.06 million goats [8]. Albeit with this huge potential, the status of bluetongue disease and vectors involved in the transmission in Ethiopia has not been well explored. This could be due to the fact that bluetongue disease is misdiagnosed with other highly prevalent ruminant diseases having similar clinical signs. However, 46.67% sheep from different agro-climatic areas of central Ethiopia [9] and 34.1% sheep of Amhara National Regional State, Northwestern Ethiopia were found seropositive to bluetongue based on competition ELISA [10]. These reports focused only on sheep and covering small agro-ecological area vis-à-vis huge livestock population and larger size of the country. Besides, virus isolation and characterization at serotype and molecular level had never been attempted. At present there is no vaccination program for control of bluetongue in small ruminants has been implemented in the country. Keeping in view of the above facts, the present study has been carried out to have a clear picture about the bluetongue disease in south western Ethiopia.

This study was the part of the bluetongue virus survey of the whole country and undertaken with the objectives to determine the seroprevalence of bluetongue in sheep and goats in selected districts in south western Ethiopia and identify the potential risk factors associated with the seroprevalence.

## Main text

### Methods

#### Study area

**Fig. 1:**
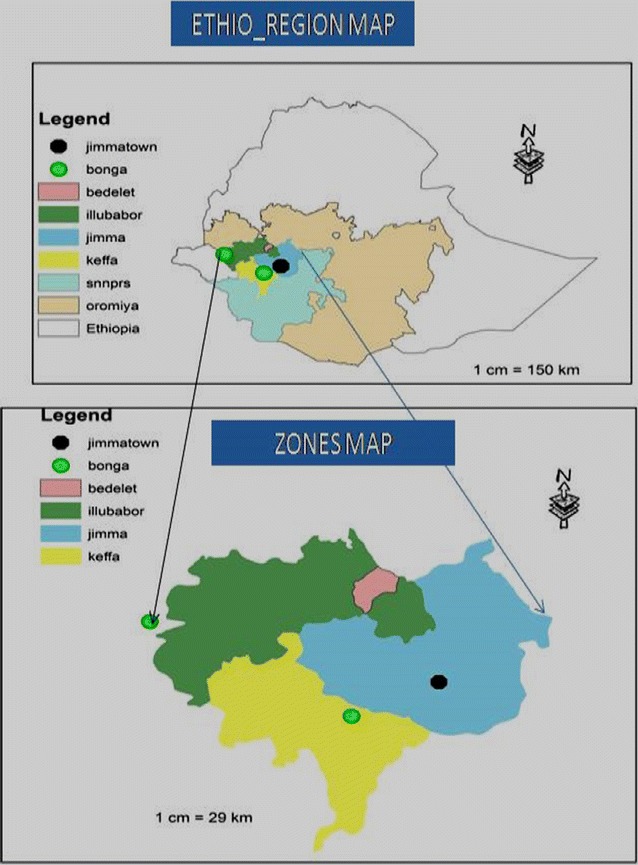
Map of study area

Study was conducted in selected districts of south western Ethiopia namely Jimma town, Bonga town and Bedele district. From each district statistically representative peasant associations (PA) were selected like Shobe, Sidisa, Qare and Yabela from Bedele, Ifa Bula, Bore and Qofe from Jimma and Bonga zuria 01 and 03 from Bonga were selected (Fig. 1).

#### Study animals

The study animals were small ruminants at different agro-ecological zones found in different peasant association of selected districts.

#### Study design and period

A cross-sectional study was conducted to estimate the seroprevalence of bluetongue virus (BTV) from December, 2015 to August, 2016 in small ruminants at south western Ethiopia.

#### Sample size determination

The sample size was determined according to Thrusfield [11] with 95% confidence interval and 5% absolute precision. There was no previous research work done in the study area so that the expected prevalence rate was taken as 50%. Therefore, 384 small ruminants were obtained for the study using the formula. However, to increase precision of the result, a total of 422 animals (248 sheep and 174 goats) were included. From each district samples were taken proportional to the total study population in the study districts of which (141 from Jimma district, 101 from Bonga district and 180 from Bedele district).

$$N = 1.96^{2} P\exp \left( {1 - P\exp } \right)/d^{2}$$

where, N = sample size, P exp = expected prevalence, and d = desired absolute precision.

#### Sampling technique

Convenience sampling was used to select study districts from different agro-ecological zones whereas simple random sampling was used to select specific animals for sampling frame and PAs. Body condition scores and age determination were done according to Vatta et al. [12].

#### Sample collection and testing

About 5 ml of blood was collected aseptically from the jugular vein of each sheep and goat using plain serum vacutainer tubes and needles. The blood was allowed to clot for 1–2 h at room temperature, stored horizontally overnight at 4 °C in the refrigerator and then the serum was separated. All the sera were stored at − 20 °C and transported on ice and submitted to the National Veterinary Institute (NVI) to screen for the presence of BTV group-specific antibodies using the Bluetongue Antibody Test Kit, c-ELISA (IDvet, 310, rue Louis Pasteur-Grabels-France) which is based on recombinant VP7 protein. The test was performed as per the manufacturer’s protocol.

#### Data management and analysis

The data collected has been uploaded in Microsoft Excel spread-sheet and analyzed using SPSS version 20.0 software. Associations between seroprevalence and its potential risk factors were tested in a univariable and multivariable logistic regression analysis. Finally, odds ratios and its 95% confidence interval (CI) were calculated and risk factors with a *P* value < 0.05 were taken as statistically significant.

### Results

In the present study, 422 serum samples from sheep (246) and goats (176) were collected and screened for the presence of BTV specific antibodies by c-ELISA. Out of 422 sera tested, 129 (30.6%) (95% CI 26.2–35) were found to be positive for BTV-specific antibodies (Table 1).

**Table 1:** Prevalence of antibodies to bluetongue virus in small ruminants from the selected PAs

Peasant association	No. tested	No. positive (prevalence %)
Bonga zuria 01	50	10 (20.0)
Bonga zuria 03	51	4 (7.8)
Bore	47	5 (10.6)
Ifa bula	47	10 (21.3)
Qare	45	14 (31.1)
Qofe	47	20 (42.6)
Shobe	45	24 (53.3)
Sidisa	47	24 (51.1)
Yabela	43	18 (41.9)
Total	422	129 (30.6)

On the basis of univariate and multivariate logistic regression analysis, it was concluded that risk factors like sex, and body condition had no significant (P > 0.05) impact (Table 2) whereas species, age, districts and agro-ecology had significant (P < 0.05) influence on the seroprevalence of bluetongue. It was found that goats were 2.3 times more likely to be positive to group specific BT virus antibody than sheep. Adults were three times compared to young animals and animals of lowland compared to highland areas were three times more likely to be seropositive to BTV respectively. Among the districts, small ruminants in Bedelle district were 2.3 times more likely to be seropositive to BTV than Jimma district (Table 2).

**Table 2:** Univariate and multivariate logistic regression analysis of bluetongue virus antibody in small ruminants based on different risk factors

Variables	Total	Prevalence (%)	P value	OR	95% CI
Species					
Sheep^a^	246	57 (23.2)	–	–	–
Goat	176	72 (40.9)	0.001	2.296	1.505–3.500
Total	422	129 (30.6)			
Sex					
Female^a^	236	74 (31.4)	–	–	–
Male	186	55 (29.6)	0.785	0.919	0.605–1.396
Total	422	129 (30.6)			
Age					
Young^a^	181	33 (18.3)	–	–	–
Adult	241	96 (39.8)	0.001	2.969	1.880–4.690
Total	422	129 (30.6)			
Body condition					
Emaciated	89	22 (24.7)	0.175	0.673	0.380–1.193
Medium	150	47 (31.3)	0.777	0.935	0.589–1.486
Good^a^	183	60 (32.8)	–	–	–
Total	422	129 (30.6)			
Altitude					
Lowland	92	48 (52.2)	0.001	5.692	3.107–10.426
Midland	187	58 (26.8)	0.002	2.346	1.363–4.038
Highland^a^	143	23 (17.7)	–	–	–
Districts					
Jimma^a^	141	35 (24.8)	–	–	–
Bonga	101	14 (13.9)	0.039	0.487	0.247–0.963
Bedelle	180	80 (44.4)	0.001	2.423	1.496–3.924
Total	422	129 (30.6)			

*OR* Normal odds ratio

^a^Reference category

### Discussions

From this study it is revealed that antibodies to BTV is highly prevalent in sheep and goats of south west Ethiopia. The results of the present study revealed the first confirmation of BTV antibody in sheep and goats from South west Ethiopia. Although, seropositivity to bluetongue virus has been reported earlier in small ruminants in Ethiopia, it has covered only a small region without mentioning the association of risk factors [9, 10, 13]. In the present study 30.6% (129/4220 serum samples of small ruminants were found positive to antibodies to BTV. This findings is in agreement with the results of Gulima [10] who reported 34.1% seropositivity to BTV in indigenous sheep of Northwestern Ethiopia. The seropositivity to BTV in small ruminants (28.6%) in India [14], domestic livestock (23%) in Kazakhstan [15], sheep in Iran (35.9%) [16], sheep in Iran (34.93%) [17], goats in Iran (39.47%) [18], small ruminants in Turkey (29.5%) [19], sheep in Iran (37.7%) [20] and again sheep in Iran (33.75%) [21] has also been reported from other parts of the world.

The present finding is found to be relatively lower than the previously reported seroprevalence to BTV in small ruminants by different authors in different countries such as 41.17% in small ruminants in Southern Ethiopia [13], 46.67% in sheep in central Ethiopia [9], 78.4% in small ruminants in Grenada [22] and 45.7% in small ruminant in India [23]. However, 30.6% seropositivity to BTV in small ruminants in the present study is comparatively higher than the reports of other researchers like 6.57% in sheep in Southeast Iran [21], 6.96% from small ruminants (13.7% in goats and 5.70% in sheep) in Algeria [24], 2.63 and 5.3% in small ruminants in Kerala and Karnataka (India) [25, 26] respectively. In the present study, the difference in the seropositivity might be due to difference in animal species, age of sampled animals, immune status of sampled animals, agroclimatic zones, ecology, and types of *culicoides* vectors.

In the current study, seropositivity to BTV in goats was higher than sheep. Higher seroprevalence among goats compared to sheep indicating that goats play an pivotal role in the epidemiology of BTV. Whereas, sheep which are highly susceptible animals to BT show distinct clinical signs and die of the disease. It is also an established fact that goats with minimum clinical manifestation maintain high titer of BTV and may be the potential source of infection to other susceptible animals [13, 14].

It has been recorded that BT virus infection is increased with increasing age of animals and this is in agreement with the reports of Yilma and Mekonnen [13]. Assessing age as a risk factor, there was a statistically significant association (P < 0.05) with the prevalence of BTV specific antibody in the study animals. It was shown that the younger animals started to get infected with BTV after the age of included at category of adult level. At this age, the animals are usually released into the pasture for grazing, where they are likely to be exposed to infection by vectors and subsequent BTV infection. Young age groups are usually kept indoors and are well taken care of by the owners from contracting infectious diseases, particularly the insect and tick-borne infections [27, 28].

This study was also assessed the effect of agro ecology on the seroprevalence of the BTV antibody and found that animals at low altitude are more prone to BTV infection than high altitude. This is in agreement with different authors [18, 22]. The prevalence correlated with the probable distribution of the *Culicoides* vector. In addition environmental changes can influence the incidence, distribution and evolution of infectious diseases, particularly those transmitted by arthropod vectors [29].

## Limitations

This manuscript is part of mega project which comprises only the serological BTV test report of the south western Ethiopia. The project is working on virus isolation, molecular characterization and identification of vectors responsible for the transmission of the disease in Ethiopia. As a limitation, this manuscript doesn’t include the molecular and virus isolation data.

## Abbreviations

BT: bluetongue
BTV: bluetongue virus
CSA: Central Statistical Authority
NVI: National Veterinary Institute
OR: odds ratio
CI: confidence interval
c-ELISA: competitive enzyme-linked immunosorbent assay
AOR: adjusted odds ratio
OD: optical density
VP: viral protein
PAs: peasant associations
SPSS: Statistical Package for Social Science
RT-PCR: reverse transcriptase polymerase chain reaction
